# Hydrolyzed Collagen—Sources and Applications

**DOI:** 10.3390/molecules24224031

**Published:** 2019-11-07

**Authors:** Arely León-López, Alejandro Morales-Peñaloza, Víctor Manuel Martínez-Juárez, Apolonio Vargas-Torres, Dimitrios I. Zeugolis, Gabriel Aguirre-Álvarez

**Affiliations:** 1 Instituto de Ciencias Agropecuarias, Universidad Autónoma del Estado de Hidalgo, Av. Universidad km 1. Ex Hacienda de Aquetzalpa. Tulancingo, Hidalgo 43600, Mexico; arlely@hotmail.com (A.L.-L.); victormj@uaeh.edu.mx (V.M.M.-J.); apolovt@hotmail.com (A.V.-T.); 2 Universidad Autónoma del Estado de Hidalgo, Escuela Superior de Apan, Carretera Apan-Calpulalpan s/n, Colonia, Chimalpa Tlalayote, Apan, Hidalgo 43920 Mexico; lacirem71@yahoo.com.mx; 3 Regenerative, Modular & Developmental Engineering Laboratory (REMODEL), National University of Ireland Galway (NUI Galway), H91 TK33 Galway, Ireland; dimitrios.zeugolis@nuigalway.ie; 4 Science Foundation Ireland (SFI) Centre for Research in Medical Devices (CÚRAM) National University of Ireland Galway (NUI Galway), H91 TK33 Galway, Ireland

**Keywords:** hydrolyzed collagen, peptide, antioxidant activity, denaturation, hydrolysates

## Abstract

Hydrolyzed collagen (HC) is a group of peptides with low molecular weight (3–6 KDa) that can be obtained by enzymatic action in acid or alkaline media at a specific incubation temperature. HC can be extracted from different sources such as bovine or porcine. These sources have presented health limitations in the last years. Recently research has shown good properties of the HC found in skin, scale, and bones from marine sources. Type and source of extraction are the main factors that affect HC properties, such as molecular weight of the peptide chain, solubility, and functional activity. HC is widely used in several industries including food, pharmaceutical, cosmetic, biomedical, and leather industries. The present review presents the different types of HC, sources of extraction, and their applications as a biomaterial.

## 1. Introduction

Collagen is the most important protein produced by the human body, it is mainly formed by the amino acid glycine (33%), proline and hydroxyproline (22%) (primary structure) in a triplex helix which is formed by three α chains. Each alpha chain is composed for 1014 amino acids approximately with a molecular weight around 100 kDa. These chains are coiled into a left-handed helix with three amino acids per turn (secondary structure). The chains are twisted around each other into a triple helix to form a rigid structure (tertiary structure). The super helix represents the basic collagen structure (quaternary structure). This collagen structure is very stable because of the intramolecular hydrogen bonds between glycine in adjacent chains. The collagen molecule is formed for a triple helical region and two nonhelical regions at either end of the helix structure with ≈300 kDa molecular weight, 280 nm in length, and 1.4 nm in diameter [1,2,3].

Nearly 28 types of collagen have been identified, but collagen type I is the most common in skin, bone, teeth, tendon, ligaments, vascular ligature, and organs. Collagen type II is present in the cartilages. For collagen type III, the skin, muscle, and blood vessels are the most common sources of this protein. Type IV has been reported in the epithelium-secreted layer of the basement membrane and the basal lamina. Collagen type V is one of the principal components of cell surfaces and placenta [4,5,6,7,8,9]. Collagens are different according to their α-chain composition, depending on the repeat and length of the Gly–X–Y amino acid repetition, with and without interruptions, also the occupation of the X and Y positions by proline and its hydroxylated form, hydroxyproline, respectively [1,10].

Collagen has been classified into different families such as fibrillar and network-forming collagens, the FACITs (fibril-associated collagens with interrupted triple helices), MACITs (membrane-associated collagens with interrupted triple helices), and MULTIPLEXINs (multiple triple-helix domains and interruptions) [11,12]. Fibrillar collagen is the most abundant collagen in vertebrates and it plays a structural role by contributing to the molecular architecture, shape, and mechanical properties of tissues such as tensile strength in skin and the resistance to traction in ligaments (collagens type I, II, III, V, XI, XXIV, and XXVII) [13,14,15].

FACITs (fibril-associated collagens with interrupted triple helices) do not form fibrils by themselves, but they are associated with the surface of collagen fibrils. FACITs collagens have their triple helix interrupted by non-collagenous domains which can act as joints. These interruptions are useful because they allow proteolytic cleavage of the structure overcoming the resistance to proteases of native triple helices [2,16]. The MULTIPLEXIN collagen family includes types XV and XVIII. Collagen types XV and XVIII represent a molecule of the basement membranes. Collagen XV is found in skeletal and cardiac muscle, and collagen type XVIII is a component in the liver. The MACIT collagen family has numerous interruptions in the triple helix, does not self-assemble into fibrils, and has roles in cell adhesion and signaling. Other collagen types are in very low concentration and in specific organs in the body [14,17].

Native collagen type I can be extracted from different sources, however, the main source of extraction is bovine because of its availability as well as its biocompatibility. Collagen extraction can be carried out from different tissues such as bones, tendons, lung tissue, or even connective tissue [18,19,20,21].

Another common source is porcine byproducts. This source has high resemblance to human collagen. There are no allergenic limitations to its usage because it has been widely used as a tendon reinforcement [22,23], hernia repairment [24], and for skin and wound healing as a plastic and reconstructive surgery material [25].

Alternative sources for native collagen extraction that are not of bovine or porcine origin have been developed from ovine tendon and skin [26,27]; fish tissue such as bones, skin, and scales or waste fish byproducts, or others sources such as chicken, duck, and rabbit skin [28,29,30,31,32].

Extraction can be carried out by an acid or alkaline treatment [33]. Extraction under acid treatment is usually applied for extraction of collagen type I from tissues of porcine or fish skin origin [34]. Acetic acid is the most common reagent for collagen extraction. The concentration of this acid will affect the final pH value changing the electrostatic interaction and structure. It also determines the solubility and extraction capacity from animal tissue [35]. A combination of both acidic and enzymatic treatment produces a higher and more efficient collagen extraction process [26]. Pepsin can be obtained from porcine gastric mucosa. This enzyme affects the telopeptidic region in the collagen molecule increasing its solubility in an acidic medium [36,37]. The use of ultrasound as an alternative method for collagen extraction does not change the molecule and facilitates the enzymatic action. This technology can be applied in different tissues such as fish skin and bovine tendons in order to produce higher collagen concentrations in shorter extraction times [38,39,40].

Pre-treatment conditions, dialysis, and source of extraction are the main factors that determine final collagen characteristics such as molecular weight, amino acid composition, and molecular structure [41,42].

## 2. Hydrolyzed Collagen: Extraction and Properties

### 2.1. Extraction and Structure of Hydrolyzed Collagen

**Figure 1:**
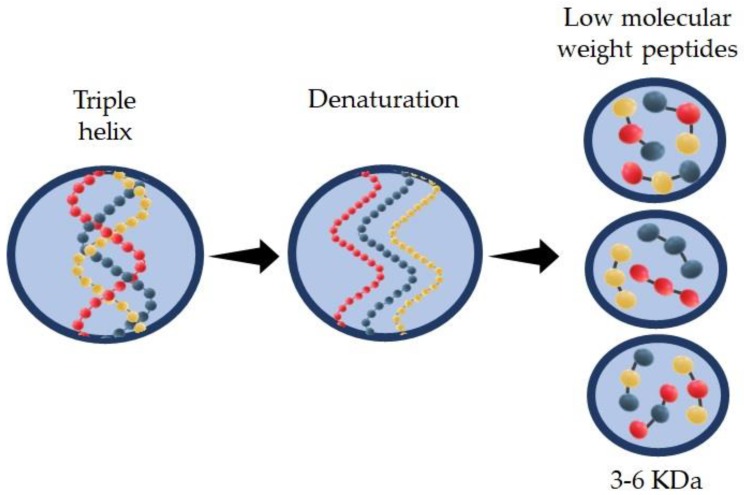
Denaturation of native collagen into small low-molecular-weight peptides.

From Figure 1, it can be seen that denaturation of native collagen produces three α chains in their random coiled form. It can be observed by thermal treatment of collagen above 40 °C. Once the chains are separated, the hydrolysis is carried out by the action of proteolytic enzymes (alcalase, papain, pepsin, and others). The resulting product is commonly called hydrolyzed collagen (HC). It is composed of small peptides with low molecular weight 3–6 KDa [43,44,45,46]. Its solubility and functional activity (antioxidant, antimicrobial) are related to the type and degree of hydrolysis as well as the type of enzyme used in the process [47,48,49,50]. Another type of hydrolysis is by use of chemical products in acidic [45,51,52,53] (acetic acid, hydrochloric acid, and phosphoric acid) or alkaline media [27,52]. These two types of extraction are strongly corrosive and produce a high salt concentration in the final product after neutralization [54]. Alternative methods of extraction consist in thermal treatment [55] or applying high temperature and pressure to the protein. It includes subcritical water level (SCW) that exists at a temperature between 100 and 374 °C and a pressure of less than 22 MPa [56,57].

### 2.2. Techniques for HC Molecular Weight Measurements

The determination of HC molecular weight is a difficult task because of its low molecular weight (Mw) which ranges between 3 and 6 KDa. The most common technique used is SDS-PAGE (sodium dodecyl sulfate polyacrylamide gel electrophoresis). It can separate proteins in the mass range of 1–100 KDa. The molecules are separated according to their charge, the moving speed is related to the charge of the molecule. This method uses polyacrylamide gels (PAGE—polyacrylamide gel electrophoresis) in the presence of the anionic detergent sodium dodecyl sulfate (SDS). The gel polymerization of acrylamide monomers produces linear chains. By including bisacrylamide, this formed a three-dimensional matrix of the gel. The size of the pores formed depends on the concentration of acrylamide and the degree of crosslinking. The first gel is the staking gel, it is a low-concentration gel (4%), and the second gel called resolving gel usually has a 10–12.5% concentration and is used to separate proteins in the range of 1–100 KDa. Thus, varying the concentration of acrylamide and bisacrylamide in the gel preparation results in different degrees of porosity and therefore different protein separation intervals [58,59,60].

Matrix-assisted laser desorption/ionization time-of-flight mass spectrometry (MALDI-TOF MS) is another technique that helps to detect molecules in a large range of molecular weights. It is a technique where peptides are first mixed with a large molar excess of a matrix compound such as DHB (2,5-dihydroxybenzoic acid) to ionize low-molecular-weight peptides, next the matrix that carries the peptides is vaporized by laser radiation, and finally the mass of vaporized peptides is determined from the ionic time-of-flight. However, the limitation of this technique is that some peptide peaks fail to resolve in a single matrix [61,62,63].

HPLC-MS/MS is a powerful tool not only for the identification, but also for quantification of peptides and proteins. It is rather limited to the quantification of selected peptides of biological importance such as the quantification of collagen. The quantification of collagen types is usually carried out by amino acid analysis [64,65,66].

### 2.3. Hydrolyzed Collagen Properties

Native collagen properties are very different to those of hydrolyzed collagen as illustrated in Table 1. After denaturation, the triple-helix structure of native collagen changes to a random coil form due to the dissociation of the hydrogen bonds when collagen suffers hydrolysis. This treatment can break the bonds in the polypeptide chain to obtain a large number of peptides. The molecular weight of collagen peptides obtained from hydrolysis is very low (3–6 KDa) compared to that of its precursor native collagen (285–300 KDa). Enzymatic hydrolysis affects not only the size of the peptides but also physicochemical and biological properties [67,68]. Viscosity is one of the physicochemical properties of collagen; the native form shows higher values due to stronger electrostatic repulsion among the molecular chains even at low concentrations of collagen solution. However, its hydrolyzed form shows very low viscosity no matter the concentration because of the low molecular weight of the small chain segments [69]. Electrostatic properties of proteins such as the isoelectric point (pI) are important parameters which are related to the proportion of acid amino residues and base amino residues in protein. Collagen is an amphoteric macromolecule that possesses a pI value between 7 and 8. On the hydrolysis process, the pI value is shifted to lower values between 3.68 and 5.7. This change will depend on the amino acid sequences and distribution of amino acid residues according to the type or time of hydrolysis [65,70,71,72]. The composition and degree of hydrolysis of collagen are factors that increase functional properties such as antioxidant capacity, antimicrobial activity, and higher bioavailability. These properties are related mainly to the molecular weight value. It makes HC to act as an electron donor to produce more stable products reacting with free radicals [73,74].

Native collagen is commonly used in different industries because it has excellent biocompatibility and biodegradability, low immunogenicity, and high versatility to fabricate films. However, HC is highly soluble in water but not able to form films by itself. It is necessary to combine it with other biopolymers [26,75]. HC presents several advantages compared to native collagen. Some of them consist in higher therapeutic loading, cost-effectiveness and not requiring a multistep extraction procedure, highly digestible and is easily absorbed and distributed in the human body [75,76]. Furthermore, it exhibits lower viscosity in aqueous solution, neutral odor, colorlessness, transparency, emulsification and stabilization, foam forming, film forming, wettability, solubility, dispersibility, powder compressibility, carrier substance and low allergenicity [65,77].

Collagen peptides can be used as an ingredient for functional food supplements because they present antioxidant and antimicrobial activity [6,78,79] and its quality depends on the methodology used to extract it [76]. These peptides have shown capacity to bind Ca^+^ ions promoting the bioavailability and making it more compatible to the human body [80,81]. HC also helps to improve memory health shown through in vivo studies, it could be a candidate ingredient of drugs used to manage and improve health [82]. In food science, HC helps to minimize or prevent damage in cells and tissues during freezer storage, therefore, it is an option to use in food that requires low-temperature storage [83].

## 3. Hydrolyzed Collagen: Sources and Applications

### 3.1. Sources

#### 3.1.1. Bovine

HC can be extracted from different sources and tissues [71], it can be extracted from bovine Achilles tendon by using different enzymes such as alcalase, pepsin, trypsin, and collagenase produced by *Penicillium aurantiogriseum*. It shows antihypertensive, antioxidant, and antimicrobial activity [85,86]. HC from bovine lung showed antioxidant and anti-inflammatory activity [87]. HC from the nuchal ligament of bovine by papain action can be used as a promising precursor of angiotensin-I-converting enzyme (ACE)-inhibitory peptides [88].

#### 3.1.2. Porcine

Another traditional source of HC is porcine skin. It presents a low molecular weight around 1–10 KDa. It is produced by a hydrothermal process and fractionated by ultra-filtration membranes; showing antioxidant, anti-aging, skin permeation properties [89], and ACE-inhibitory potency [90]. HC from porcine skin contains functional peptides commonly used in dietary supplements [91]. HC porcine extraction can be carried out by treatments that include high temperature (150–250 °C) and pressure (350–3900 KPa). These parameters of extraction generate peptides with lower molecular weight than 15 KDa [92].

#### 3.1.3. Marine

HC extraction from traditional sources such as porcine and bovine involves some limitations due to health problems such as swine flu [93] and bovine spongiform encephalopathy [94]. Moreover, religious issues must be included [95]. Researchers have been focused on the development of a new source of extraction. Alternative sources have been investigated from marine sources such as fish and other invertebrates such as jellyfishes or sponges [25,96,97]. HC from *Prionace glauca* extracted with alcalase enzyme hydrolysis reported peptides with molecular weight lower than 20 KDa and nutraceutical effects [98]. Tilapia scales (*Oreochromis niloticus*) have been used to produce HC with high quality and low Mw [99]. HC extracted from pacu and rohu waste by using collagenase Type I from *Clostridium*, showed molecular weight hydrolysates of around 5 KDa. It was used as a peroxide inhibitor in lipid-based food and cytoprotective agent in cell culture [100]. Marine byproducts such as fish viscera also represent a good source for extraction of HC. This waste material has been used to produce HC with functional bioactive properties [101]. However, by changing the extraction parameters from different temperatures (150–300 °C), pressure (50–100 bar), and reaction time (5 min), it is possible to obtain HC from tuna skin with low molecular weight (<600 Da) and antioxidant and antimicrobial activity [102].

Other marine sources for preparation of HC were obtained from cod protein hydrolysate (*Gadus morhua*) [103], Alaska pollock [104], and cartilage of spotless smooth hound [105]. The extracted biomaterials results were lower molecular weights (3–5 KDa) and antioxidative activity.

#### 3.1.4. Alternative Sources

Some alternative sources present great functionality properties. HC extracted from chicken legs by enzymatic action (proteases) [106] and skin of *Rana chensinensis* by acid hydrolysis [107] exhibited high solubility, angiotensin-converting enzyme inhibition, and antioxidant activity. Chicken feet treated with papain enzymes at different temperatures (4, 30, and 56 °C) and different extraction times (20, 24, and 28 h) showed functional properties such as water and oil retention capacity as well as emulsifying and foaming properties [108,109].

### 3.2. Applications

The molecular weight and functional properties of HC depended on the source, type of extraction, and type of enzyme used during extraction. These properties could help determine the applications of HC in cosmetic, pharmaceutical, biomaterials, food, and nutraceutical industries [101,110,111].

#### 3.2.1. Oral Collagen Supplementation

Collagen loss in the body starts at 18–29 years of age, after 40 years the human body can lose around 1% per year, and at around 80 years collagen production in the body can decrease 75% overall in comparison to that of young adults [112,113]. There are other factors contributing to this such as free radicals in the organism, deficient diet, smoking, alcoholism, and disease. The role of collagen in the body is very important because it helps the development of the organs; wound and tissue healing; cornea, gums, and scalp repair. Collagen helps in bone and blood vessel reparation. In the cornea, collagen tissue gets mechanical and optical properties. It is present in biological functions of the cell such as proliferation, cell survival, and differentiation; so collagen is present in the human body as a whole in bones, tendons, ligament, hair, skin, and muscles [2,114,115].

The skin is the largest organ in the human body, collagen elastic fibers and hyaluronic acid are its major structural constituents. Skin protects the organism from external damages, regulates temperature, and performs other bodily functions [116,117,118]. Aging is a natural process which involves changes in the human body; the skin suffers morphologic, structural, and functional deterioration; collagen reduces and elastin fibers promote the formation of lines and wrinkles. Skin aging control is a challenge in the cosmetic industry, but HC has proved to be an alternative solution in slowing down the effects of aging [74,110,119,120]. HC from milkfish scales exhibited excellent water-holding capacity, moisture absorption, and retention as well as anti-skin aging, and anti-melanogenic capacities, proving a potential active ingredient in skin care products [121].

Hydrolyzed collagen acts in two different forms in the dermis; in the first action, the free amino acids provide building blocks for the formation of collagen and elastin fibers. In the second action, collagen oligopeptides act as ligands, binding to receptors on the fibroblasts’ membrane and stimulating the production of new collagen, elastin, and hyaluronic acid [76].

In recent years, oral collagen supplementation has become popular as it has been increasingly marketed to consumers as an anti-aging product, because HC oral supplementation reaches the deeper layers of the skin and improves skin physiology and appearance increasing hydration, elasticity, firmness, wrinkle reduction, and skin rejuvenation [122,123] (Table 2).

Studies in vivo in woman between 40 and 60 years of age with HC oral supplementation during 12 weeks, showed a significant improvement in skin hydration, wrinkling, and elasticity [117]. HC as an oral nutrient supplement in women between 35 and 65 years of age proved after three months enhancement in dermal thickness, skin firmness, and elasticity after treatment [124]. Another study with 120 subjects during 90 days, where they consumed daily oral supplementation of a liquid nutraceutical containing fish HC, resulted in improvement of skin texture and elasticity. Additionally, a protective effect on joint health was observed [119]. Oral supplementation of HC in women between 40 and 59 years of age revealed a significant increment in skin hydration and collagen density at dermis level. However, fragmentation of the dermal collagen network significantly decreased after four weeks of supplementation and these effects persisted after 12 weeks [125].

A study of sixty healthy female subjects, aged between 40 and 50 years, after 28 days of oral supplementation showed that this acted on skin elasticity and presented a more pronounced effect on dermis echogenicity, reducing skin pores, improving hydration, texture, elasticity, and firmness of the skin. The oral supplementation product was composed of HC with a mix of vitamins A, C, E, and zinc [126]. HC from tilapia fish scale (*Oreochromis mosambicus*) in the form of 20 mL beverage (Nitta Gelatin Inc^®^., India) was consumed by healthy women aged 30–60 years over 12 weeks, after this time the result was enhanced facial skin moisture [127]. HC drinking ampoules with fruit extract, Vit C, E, zinc, and biotin ELASTEN^®^ were supplied to 36 healthy women aged 35 years or older for 12 weeks and their skin showed higher hydration, elasticity, smoothness and density [123].

#### 3.2.2. Food Industry

HC presents antioxidant and antimicrobial activity, so it can be used as a functional ingredient in food supplements as well [6,78,79]. Collagen hydrolysates can attach calcium ions, improving its bioavailability, therefore, HC can be used in functional food ingredients in the management of mineral deficiencies [80,81]. HC acts as an anticoagulant because it helps to decrease the damage in cells and tissues originated by low temperatures, therefore, it could be useful in foods that require storage in cold or freezing temperatures [128]. HC has been used in the preparation of different products such as meat products, beverages, soups, and others. It helps increase and maintain their sensorial, chemical, and physical properties.

HC has been used in processed foods such as sausages to replace pork fat at 50% level of replacement. The final product results had greater water holding capacity, better stability after cooking, and improved texture such as hardness and chewiness [129]. The use of fish HC in meat products such as buffalo patties, resulted in higher protein content, lower fat content, similar sensory acceptability, and better texture as compared to the buffalo patties without HC [130]. HC from bovine skin was used in combination with modified starch and guar gum in ham elaboration. Lower syneresis with 2.0% of HC final concentration in the product was reported as the best treatment [131].

HC from fish can be added in beverages such as orange juice (2.5%), and the product showed an improvement in nutritional and functional properties with a higher protein content, bioavailability, and low viscosity as well as high solubility in water [48]. The development of a fermented dairy drink using ricotta cheese whey with HC added as a functional ingredient presented low syneresis and sedimentation, with good physical–chemical and microbiological properties [132]. Dairy beverages using HC, açaí pulp, and cheese showed higher sensorial acceptability, affecting positively the viscosity and presenting adequate physicochemical and microbiological parameters after 28 days of storage [133].

HC can be added in soup as well, it has an effect in its viscosity and the functional properties. It presented high 2,2′-azino-bis-3-ethylbenzothiazoline-6-sulphonic acid (ABTS) and 2,2-diphenyl-1-picryl-hydrazyl (DPPH) radical scavenging activities [134]. Addition of HC from pigskin shavings (collagen waste) in a chrysanthemum beverage, showed an excellent clarification effect, better sensorial quality, and storage stability. However, the amount of HC added as a clarifier was lower compared to that of other commercial clarifiers [135] (Table 3). HC from pig skin extracted by enzymatic action exhibited high flocculation capability under acidic and neutral conditions, this property could be caused by the synergistic effect of optimal molecular weight distribution and electric charge [136]. Additionally, HC has been used in different food products to develop their physicochemical and functional properties. This makes HC one of the most promising functional ingredients because it does not affect sensorial properties.

#### 3.2.3. Biomaterials

Collagen presents good biocompatibility and biodegradability, hence, it is safe and effective as a biomaterial, it has been used in the last years as a safe and effective biomaterial in tissue engineering and clinical applications [75,137,138].

Compared to native collagen, HC has a main advantage—it presents higher solubility; moreover, HC extraction is simple and does not require a multistep extraction procedure [75,139,140]. However, HC cannot form scaffolds by itself because of the low molecular weight of the peptides, but it can be mixed with other biopolymers such as cellulose and chitosan.

Films prepared with a blend of cellulose–HC exhibited good transparence, good ultraviolet radiation absorption, and excellent support for cell adhesion and proliferation. High biocompatibility dictates that the films would have promising applications in the biomaterial field [139]. Collagen–HC films developed from leather waste were very transparent and had excellent barrier properties against UV light and studies such as FTIR and differential scanning calorimetry (DSC) showed total miscibility between both polymers [141].

The development of a HC collagen biomaterial could be beneficial for the management of bone and joint disorders because of HC’s low molecular weight and amino acid composition. It is more bioavailable and induces a better osteointegration by promoting collagen synthesis [140].

Alternative biomaterials with HC are chitosan sponges. They are prepared by sol–gel transition methodology showing porous morphology, improved biostability, good water uptake capacity, excellent biocompatibility, and antimicrobial activity by the presence of HC [75].

HC has been used in hydrogel development for the delivery of drugs such as insulin and methylene blue, showing lower water absorbency. The hydrogel delivery was faster at pH 2. It is useful for drugs susceptible to degradation under the acidic and proteolytic environment of gastric fluids [142]. Hydrogels prepared with chitosan and fish HC showed antibacterial activity against *Escherichia coli*, *Staphylococcus aureus*, pro-cell proliferation and migration, and wound healing efficiency [143]. Nanofibrous scaffolds can be functionalized with HC as a regenerative component using electrospinning methodology. It presented porous morphology, adequate mechanical strength, excellent biocompatibility, and antimicrobial properties against *E. coli* and *Pseudomonas aeruginosa* [144] (Table 4).

## 4. Future Trends

Hydrolyzed collagen can be obtained from agro-food waste (bones, tendons, and skin). The recycling of these byproducts can help reduce the pollution generated by these types of wastes, transforming them into a new product with a high functional value. Traditionally, denaturation of collagen has been carried out with acids or alkaline products; however, the application of emergent technologies such as the combination of high temperatures and pressures as well as high intensity ultrasound have been investigated in order to decrease the disposal of chemical products. Low-molecular-weight peptides could be applied into food systems, for example, beverages with HC show a great advantage of easy digestion, high assimilation (about 80%), and good absorption at the intestinal level. It would be important to develop drinks with HC considering they will have functional properties for consumption in most people due to its null toxicity and zero allergenicity [111,145].

## 5. Conclusions

HC has a wide range of applications due to its properties such as: low viscosity in aqueous solutions, neutral odor, colorlessness, transparency, emulsification and stabilization, foam forming, film forming, wettability, solubility, dispersibility, powder compressibility, substance carrier of low allergenicity as well as antioxidant and antimicrobial activity. For cosmetic applications, some studies have shown that HC has good biological functions such as increment of cell proliferation, water-holding capacity, moisture absorption, retention, and anti-aging in skin. HC is widely used as a functional ingredient in the food industry because of its properties to increase water holding in meat products, sensorial development, and improvement of chemical and physical properties in beverages and dairy products. Within the biomedical industry, the application of HC blended with cellulose or chitosan for the preparation of scaffolds has helped with promotion of collagen synthesis, management of bone and joint disorders, wound treatment, excellent biocompatibility, and antimicrobial properties.

## Figures and Tables

**Table 1:** Properties of native and hydrolyzed collagen.

Properties	Type of Collagen		Reference
	Native	Hydrolyzed	
Molecular weight (Mw)	~300 KDa	3–6 KDa	[65,68]
Isoelectric point (pI)	7.0–8.3	3.68–5.7	[72,84]
Viscosity	High	Low (0 Cp)	[27,69]
Film formation	Yes	No	[26,75]

**Table 2:** Hydrolyzed collagen (HC) applications as functional supplementation food with positive effect on the skin.

Product	Source	Functionality	Reference
HC from fish	Milkfish scales	Excellent water-holding capacity, moisture absorption, and retention and anti-skin aging, and anti-melanogenic capacities.	[121]
HC oral supplementation	Sutchi catfish skin	Improved skin hydration, wrinkling, and elasticity in women aged 40–60 years for 12 weeks.	[117]
HC oral nutrient supplement	Delphynol^®^	Improved dermal thickness, skin firmness, and elasticity in women between 35–65 years old for three months.	[124]
Fish HC oral supplementation	Fish	Improved skin texture and elasticity and in addition a protective effect on joint health in 120 subjects for 90 days.	[119]
HC supplementation	Fish	Increased skin hydration and collagen density in the dermis in women between 40 and 59 years old.	[125]
HC oral supplementation with vit A, C, E and zinc.	Not defined by the author	Improved the hydration, texture, elasticity, and firmness of the skin in women between 40 and 50 years after 28 days of treatment.	[126]
HC beverage	Nitta Gelatin Inc ^®^	Changes in periorbital wrinkles, facial skin hydration, and skin elasticity in healthy women aged 30–60 years.	[127]
HC drinking ampoules with acerola fruit extract, vitamin C,E zinc, and biotin	ELASTEN^®^	12 weeks oral supplementation improved the skin properties such as: hydration, elasticity, roughness, and density.	[123]

**Table 3:** Hydrolyzed collagen in the food industry.

Product	Source	Functionality	Reference
HC in sausages to replace pork backfat	Germina^®^	Sausages with greater water holding capacity and improved texture	[129]
Fish HC in buffalo patties	Az-Zahrah Sdn. Bhd^®^	Product with higher protein content, lower fat content	[130]
Ham with bovine HC-modified starch and guar gum	Bovine skin	Ham with lower syneresis	[131]
Fish HC in orange juice	Fish	Higher protein and high solubility in water, low viscosity	[48]
Fermented dairy drink	Luchebras^®^	Low syneresis and sedimentation, with good physical–chemical and microbiological properties	[132]
Dairy beverage with HC, açaí pulp, and cheese	Tovani Benzaquen Ingredients^®^	High sensory acceptability, good physicochemical and microbiological parameters	[133]
HC in herbal soup	Seabass skin	High antioxidant activity by radicals ABTS and DPPH inhibition	[134]
Chrysanthemum beverage	Pigskin	Excellent clarification effect and better sensory quality and storage stability	[135]

**Table 4:** Hydrolyzed collagen biomaterial applications.

Product	Source	Functionality	Reference
Cellulose–HC films	Jinjian Gelatin Co. Ltd.^®^	Capacity for ultraviolet radiation absorption and good support fir cell adhesion and proliferation, showing good biocompatibility	[139]
Collagen–HC films	Wastes by leather industries	Excellent barrier properties against UV light	[141]
HC biomaterial	Calf pelt	Beneficial for management of bone and joint disorders	[140]
HC-chitosan sponges	Bovine Achilles tendon	Good water uptake capacity and excellent biocompatibility and antimicrobial activity	[75]
HC hydrogels	Udomkorn Engineering Co., Ltd.^®^	Low water absorbency and faster drug release in acid pH	[142]
HC-chitosan hydrogels	Tilapia fish	Antibacterial activity against *Escherichia coli* and *Staphylococcus aureus*	[143]
Nanofibrous scaffold functionalized with HC	Tilapia fish	Antimicrobial property against *E. coli* and *Pseudomonas aeruginosa*	[144]
